# Exploring Antimicrobial Resistance in Agents Causing Urinary Tract Infections at a Tertiary Care Hospital in a Developing Country

**DOI:** 10.7759/cureus.9735

**Published:** 2020-08-14

**Authors:** Zuhair Ali Rizvi, Ali Murad Jamal, Ali Hassan Malik, Syed Muhammad Jawad Zaidi, Naimat Ullah Abdul Rahim, Daneyal Arshad

**Affiliations:** 1 Surgery, Rawalpindi Medical University, Rawalpindi, PAK; 2 Urology, Rawalpindi Medical University, Rawalpindi, PAK

**Keywords:** urinary tract infections, antimicrobial resistance

## Abstract

Background and objective

Urinary tract infections (UTIs) are usually treated with empirical therapy by physicians based on previous knowledge of the predictability of causative agents and their antimicrobial susceptibilities. The objective of this study was to determine the frequency of various pathogens causing UTIs and their antimicrobial resistance profile in patients presenting to the outpatient department (OPD) of a tertiary care hospital.

Materials and methods

This descriptive cross-sectional study was conducted in the urology OPD of a tertiary care hospital in Pakistan. The study was conducted over a period of six months, and it included 1,000 patients (of ages 12 years or above) who were clinically suspected for UTIs. Patients with comorbidities and immunocompromised patients were excluded from the study. Recipients of corticosteroid therapy or those with a history of intake of broad-spectrum antibiotics in the previous 15 days were also excluded. The modified Kirby-Bauer disc diffusion method was used for determining antimicrobial resistance against various antimicrobials.

Results

Out of 1,000 tested specimens, 530 (53%) isolates were found to be culture-positive. E.coli was the most common species isolated from the cultures with a prevalence of 77.4%, followed by Klebsiella (6.4%), Enterobacter (6.0%), Pseudomonas (3.8%), Staphylococcus saprophyticus (3.4%), Citrobacter (1.1%), and Morganella (0.4%). Antimicrobial resistance against commonly used antimicrobials was found to be alarmingly high.

Conclusion

E.coli was the most commonly isolated microorganism from the urine samples of UTI patients. Antimicrobial resistance against UTI-causing organisms is of great concern. The Surveillance of trends of antimicrobial susceptibility pattern for organisms causing UTIs is highly important. Antibiotics should be prescribed according to proper guidelines to prevent increasing antimicrobial resistance.

## Introduction

Urinary tract infections (UTIs) are one of the most commonly diagnosed diseases in outpatient departments (OPD) [1]. The selection of antibiotic therapy by a physician to treat UTI is based on the knowledge of prevalent microorganisms, recent updates about the antimicrobial susceptibility patterns, and the clinical status of the patient [2]. Studies have shown that E.coli is the most commonly isolated microorganism from UTI patients with a varying prevalence ranging from 26 to 55% [3,4].

There is conflicting data in the literature pertaining to the antimicrobial susceptibility patterns of UTI-causing organisms. A study conducted in Ethiopia showed a varying spectrum of antibiotic sensitivity pattern with 93.3% of the isolates being sensitive to gentamicin; however, less than 60% of the isolates were sensitive to chloramphenicol, nitrofurantoin, ciprofloxacin, trimethoprim-sulfamethoxazole (TMP-SMX), ceftriaxone, and nalidixic acid [5]. Another study showed a relatively lower susceptibility towards these antimicrobial agents, suggesting an increase in resistance towards these antibiotics [6]. The situation continues to worsen, especially in developing countries where the lack of appropriate surveillance and improper use of antibiotics contribute towards increased resistance in UTI-causing microorganisms [5,6].

Therefore, there is a need to conduct a study to determine the prevalence of most common agents associated with UTIs and their current antimicrobial resistance patterns, in order to formulate better antimicrobial therapies. Thus, our study aims to determine the frequency of agents causing UTI and their current antimicrobial resistance profiles. We believe this will contribute to the existing knowledge about antimicrobial resistance and ultimately improve treatment modalities for patients suffering from UTIs.

## Materials and methods

This descriptive cross-sectional study was conducted among patients presenting to the urology OPD of Benazir Bhutto Hospital, Rawalpindi, Pakistan from January 2017 till June 2017. Patients presenting to the OPD with typical clinical symptoms (burning micturition, fever, and pelvic pain) of UTI were included in the study. Patients with comorbid conditions including diabetes mellitus, renal pathologies, immunodeficiency disorders, malignancies, and congenital urogenital disorders were excluded based on their history. Patients receiving corticosteroid therapy or broad-spectrum antibiotics for the previous 15 days were also excluded from the study. The study was granted ethical approval by the Institutional Review Board of Rawalpindi Medical University. Consent was obtained from all participants and confidentially was maintained. Patients who did not give consent or refused to be a part of the study were also excluded.

Keeping in mind the exclusion criteria, the subjects were provided with a wide-mouthed standard-sized sterile container. They were advised to clean the area around urethra with water before collecting the sample, let the area dry, and collect the sample by catching it mid-stream with the container being held at 2-3 inches away. 50 μl of urine was taken on a clean slide and a coverslip was placed on it. The slide was then viewed under a microscope. The presence of blood cells, epithelial cells, pus cells, or cast bodies was duly noted. The presence of 10 or more pus cells per high-power field was considered significant pyuria. The gram-staining technique was employed. Detection of at least one or more morphologically similar bacteria per oil immersion field was considered significant.

10 µL of the specimen was transferred to a MacConkey Agar plate using a calibrated loop method. The agar plates were incubated at 35-37 °C for 24 hours for the identification of lactose-fermenting and non-lactose-fermenting bacteria. A specimen was considered positive for UTI if a single organism was cultured at a concentration of 105 colony-forming units/ml. Cysteine lactose electrolyte deficient (CLED) medium was used for the identification and isolation of urinary pathogens.

The modified Kirby-Bauer disc diffusion method was used for determining the antimicrobial susceptibility. The colonies were placed on agar plates using a sterile inoculating wire loop. Antibiotic disks were placed using sterile forceps. The plates were left for one hour at room temperature to allow for the diffusion of antibiotics from the disks. The agar plates were again incubated for 24 hours at 37 °C. Antimicrobial susceptibility and resistance were tested against ampicillin, amoxicillin+clavulanic acid (AMC), gentamicin, amikacin, cefoperazone, ceftazidime, cefixime, ceftriaxone, cefepime, piperacillin+tazobactam (TZP), cefoperazone+sulbactam (CFP+SUL), carbapenem, fosfomycin, trimethoprim/sulfamethoxazole (SXT), ciprofloxacin, ofloxacin, levofloxacin, norfloxacin, and nitrofurantoin.

The frequencies and percentages of various microorganisms isolated from the urine samples were tabulated. The antimicrobial resistance profiles of the isolated organisms were expressed in percentages. The data were analyzed using SPSS Statistics version 23 (IBM, Armonk, NY).

## Results

Out of 1,000 clinically suspected UTI patients, 530 (53%) were found to be culture-positive, while 470 (47%) were culture-negative. E.coli was the most frequently isolated microorganism, followed by Klebsiella spp., Enterobacter spp., Pseudomonas spp., Staphylococcus spp., Proteus spp., Citrobacter spp., and Morganella spp. The frequencies and percentages of different isolated microorganisms across gender are delineated in Table 1.

**Table 1:** Frequencies and percentages of various isolated microorganisms from urinary samples

Isolated microorganism	Total patients (n=530)	Males (n=160)	Females (n=370)
E.coli	410 (77.4%)	116 (72.5%)	294 (79.5%)
Klebsiella spp.	34 (6.4%)	10 (6.2%)	24 (6.5%)
Enterobacter spp.	32 (6%)	4 (2.5%)	28 (7.6%)
Pseudomonas spp.	20 (3.8%)	14 (8.8%)	6 (1.6%)
Staphylococcus spp.	18 (3.4%)	10 (6.2%)	8 (2.2%)
Proteus spp.	8 (1.5%)	4 (2.5%)	4 (1.1%)
Citrobacter spp.	6 (1.1%)	2 (1.2%)	4 (1.1%)
Morganella spp.	2 (0.4%)	0	2 (0.5%)

The isolated microorganisms had a varying spectrum of antimicrobial resistance against a plethora of antibiotics. This is elucidated in Table 2.

**Table 2:** Antimicrobial resistance profile of various UTI-causing microorganisms against commonly used antibiotics The values are expressed in percentages UTI: urinary tract infection; AMC: amoxicillin+clavulanic acid; TZP: piperacillin+tazobactam; CFP+SUL: cefoperazone+sulbactam; SXT: trimethoprim/sulfamethoxazole

Antimicrobial agent	E. coli	Klebsiella spp.	Enterobacter spp.	Pseudomonas spp.	Staphylococcus spp.	Proteus spp.	Citrobacter spp.	Morganella spp.
Ampicillin	87.3	100.0	50.0	0.0	100.0	100.0	100.0	100.0
AMC	62.7	100.0	50.0	0.0	0.0	2.0	100.0	100.0
Gentamicin	31.0	43.8	86.7	40.0	12.5	33.3	66.7	0.0
Amikacin	1.0	25.0	0.0	20.0	0.0	33.3	0.0	0.0
Cefoperazone	100.0	100.0	100.0	20.0	0.0	100.0	0.0	0.0
Ceftazidime	100.0	100.0	100.0	22.2	0.0	50.0	0.0	0.0
Cefixime	66.0	50.0	66.7	91.7	0.0	25.0	0.0	0.0
Ceftriaxone	63.7	47.1	66.7	100.0	0.0	25.0	0.0	0.0
Cefepime	63.2	43.8	66.7	22.2	0.0	0.0	0.0	0.0
TZP	11.3	17.6	33.3	22.2	0.0	0.0	66.7	0.0
CFP+SUL	10.3	29.4	33.3	22.2	0.0	0.0	66.7	0.0
carbapenem	2.5	5.9	16.7	22.2	0.0	33.3	0.0	0.0
Fosfomycin	2.0	0.0	20.0	0.0	33.3	100.0	33.3	100.0
SXT	71.9	52.9	66.7	33.3	33.3	25.0	66.7	0.0
Ciprofloxacin	75.4	47.1	87.5	60.0	0.0	0.0	33.3	0.0
Ofloxacin	100.0	100.0	100.0	70.0	0.0	0.0	0.0	0.0
Levofloxacin	93.3	88.9	100.0	77.8	75.0	50.0	0.0	0.0
Norfloxacin	75.4	40.0	0.0	70.0	42.9	33.3	66.7	0.0
Nitrofurantoin	10.7	70.6	0.0	66.7	0.0	100.0	33.3	100.0

## Discussion

UTIs are a very common problem and represent a major burden on healthcare systems, especially in developing countries [6]. Our study showed a plethora of organisms causing UTIs with varying patterns of resistance against many broad-spectrum antibiotics. UTIs are becoming difficult to treat owing to increasing resistance and intensifying global disease burden [7,8]. The emergence of resistance against broad-spectrum antibiotic agents such as extended-spectrum beta-lactams, fluoroquinolones, and carbapenem is causing problems worldwide [9].

E.coli was the most commonly isolated pathogen from our tested urine samples with a high prevalence of 77.4%. This overwhelming presence of E.coli was also observed in another study with 53.6% samples being culture-positive for E.coli, 14.6% for Proteus, 13.9% for Klebsiella, 4.5% for Enterococcus, and 4.1% for Staphylococcus [10]. In another study, it was observed that 60% of isolates from urinary samples were of E.coli, 12% of Klebsiella, and 8% of Enterococcus [11]. Similarly, a study conducted in India indicated an alarmingly high prevalence of 69.8% for E.coli, followed by Klebsiella (7.9%), Staphylococcus (4.8%), and 1.6% for methicillin-resistant Staphylococcus aureus (MRSA) [12]. The results obtained from these studies are comparable to our observations, indicating very similar trends in the prevalence of various causative organisms for UTI.

The antimicrobial resistance profile of E.coli in our study showed that more than 75% of the strains were resistant to fluoroquinolones; however, the resistance profile towards more broad-spectrum antibiotics including nitrofurantoin, fosfomycin, and carbapenem ranged between 2-10.7%. Another study indicated an alarming resistance rate of E.coli towards broad-spectrum fluoroquinolones with 60% of strains being resistant to them [4]. The resistance patterns of E.coli in our study were also similar to another study that observed an 81.8% resistant strains of E.coli to ampicillin, 54.5% to cefepime, 2.3-6.8% to carbapenem, 36.4% to gentamicin, 9.1% to amikacin, up to 66% for fluoroquinolones, and 15.9% to nitrofurantoin [12]. Another study reported that 40.5% strains of Klebsiella were resistant to gentamicin, 5.4% to carbapenem, 78.4% to levofloxacin, and 83.8% to nitrofurantoin, while according to our observations, resistance rates were 43.8%, 5.9%, 88.9%, and 70.6%, respectively [13]. Another study showed a similar resistance profile of E.coli against ampicillin with 69.5% of strains being resistant to it [14]. A study conducted in the United States indicated that the resistance among isolates of E.coli from urinary samples was lowest for nitrofurantoin (<1%) [15]. This finding was quite in contrast with our observation where the resistance profile of E.coli against nitrofurantoin was found to be 10.7%. However, another study reported the resistance rate of E.coli against nitrofurantoin to be around 15%, which is consistent with our findings [16].

The increasing resistance of E.coli towards broad-spectrum antibiotics like nitrofurantoin is of great concern and demands the need for appropriate antibiotic use according to the recommended guidelines. As there is no strict adherence to antibiotic stewardship, the clinicians tend to prescribe broad-spectrum antibiotics that contribute to increased antimicrobial resistance [17,18]. Researchers have recommended conducting quality improvement programs to ensure appropriate antibiotic use according to the proposed guidelines, especially in a developing country like ours. The increased resistance of common microorganisms causing UTI is of huge concern for the clinicians as providing broad-spectrum alternatives can predispose the patients towards their horrendous adverse effects. Adequate control on antibiotic prescription according to proper guidelines will aid in the delivery of top-quality patient care and prevent the development of multidrug-resistant organisms.

This article has been posted on a preprint server (bioRxiv) [19]. The preprint version is not pending full publication elsewhere.

## Conclusions

E.coli is the most commonly isolated pathogen from urine samples of clinically suspected UTI patients. Antimicrobial resistance in UTI-causing agents against commonly used antimicrobials is extremely frightening. The alarming antimicrobial resistance profile of UTI-causing organisms strongly indicates the need to establish proper guidelines for the use of antibiotics in the treatment of UTI patients. We hope this study opens up new horizons for advanced quality improvement projects and further research on appropriate antibiotic prescription and usage.
